# Current Status of Chronic Intestinal Failure Management in Adults

**DOI:** 10.3390/nu16162648

**Published:** 2024-08-10

**Authors:** Héctor Solar, Mariana L. Ortega, Gabriel Gondolesi

**Affiliations:** Nutritional Support, Intestinal Rehabilitation and Intestinal Transplant Unit, Hospital Universitario Fundación Favaloro, Buenos Aires C1093AAS, Argentina; maortega@ffavaloro.org (M.L.O.); ggondole@ffavaloro.org (G.G.)

**Keywords:** intestinal failure, parenteral nutrition, intestinal rehabilitation, intestinal surgery

## Abstract

Background: Chronic intestinal failure (CIF) is a heterogeneous disease that affects pediatric and adult populations worldwide and requires complex multidisciplinary management. In recent years, many advances in intravenous supplementation support, surgical techniques, pharmacological management, and intestinal transplants have been published. Based on these advances, international societies have published multiple recommendations and guidelines for the management of these patients. The purpose of this paper is to show the differences that currently exist between the recommendations (ideal life) and the experiences published by different programs around the world. Methods: A review of the literature in PubMed from 1980 to 2024 was carried out using the following terms: intestinal failure, CIF, home parenteral nutrition, short bowel syndrome, chronic intestinal pseudo-obstruction, intestinal transplant, enterohormones, and glucagon-like peptide-2. Conclusions: There is a difference between what is recommended in the guidelines and consensus and what is applied in real life. Most of the world’s countries are not able to offer all of the steps needed to treat this pathology. The development of cooperative networks between countries is necessary to ensure access to comprehensive treatment for most patients on all continents, but especially in low-income countries.

## 1. Introduction

Intestinal failure (IF) is a condition that affects both adult and pediatric populations, which not only impacts intestinal functions but also influences other organs; it is defined as the “reduction of gut function below the minimum necessary for the absorption of macronutrients, water and electrolytes requiring the use of parenteral support in the form of intravenous fluid and electrolytes (FEs) or parenteral nutrition (PN) to maintain health and/or growth” [1]. This definition has been utilized to classify the condition in the current 11th revision of the International Classification of Diseases (ICD-11) by the World Health Organization (WHO), under code DA.96.05, parent of code 96.0 on intestinal malabsorption [2].

**Figure 1:**
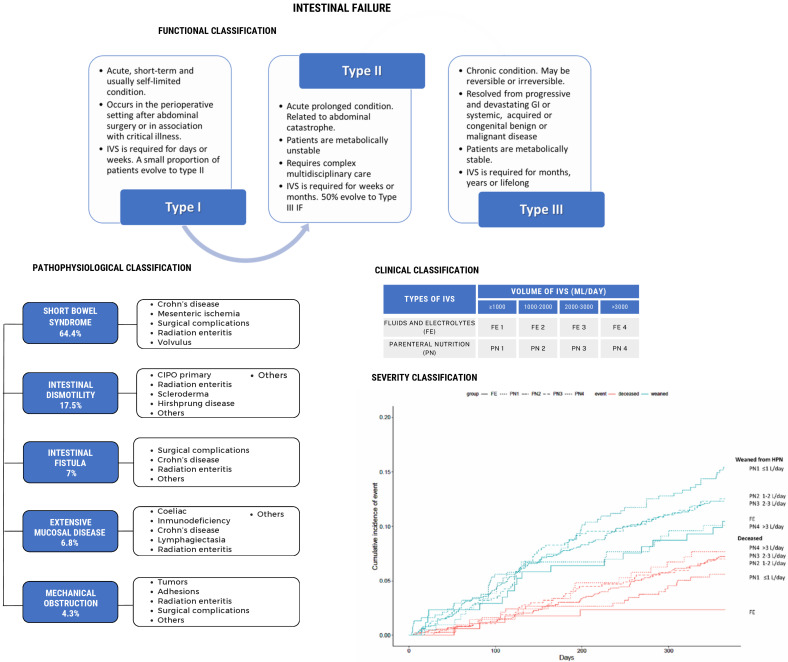
Functional, pathophysiological, clinical, and severity classification of IF.

IF is a complex and debilitating disease; its complexity is evidenced by the following facts: (a) from 1981 to 2020 was the time required to obtained a consensus on the final and current definitions and classification of the disease; (b) the etiology is multifaceted; (c) the functional classification divides IF into three types; (d) according to the pathophysiological classification, there are five major mechanisms; (e) the clinical classification (applied only to chronic intestinal failure: CIF) consists of eight categories and can be used to classify the severity of CIF (Figure 1) [3,4,5]. This is a disabling disease because individuals with CIF secondary to short bowel syndrome (CIF-SBS) or intestinal fistula have stomas, fistulas, tubes, and catheters that cause skin lesions, pain, discomfort, and body image alterations. The necessity for additional surgeries to restore intestinal continuity provokes anxiety and a fear of encountering new complications. Additionally, intestinal dysmotility, extensive small bowel mucosal disease, or intestinal obstruction can be secondary to chronic diseases, most of them without a specific treatment, requiring chronic care and home intravenous supplementation (HIVS) for months or years, and sometimes lifelong. The long-term dependence of HIVS is a characteristic of CIF patients and is associated with life-threatening complications that will lead patients to require an intestinal or multivisceral transplant (ITx or MVTx) as the last alternative to recover intestinal autonomy. All of these factors impair IF patients’ and their families’ quality of life (QoL) and impose high costs on the health system.

Currently, there are few reports on the management and results of acute intestinal failure (AIF), possibly because most AIF cases are handled by clinicians or surgeons in intensive care or surgical units, and not all are diagnosed, treated, and reported as IF patients. The majority of the information published and the greatest medical and surgical advances have occurred in CIF-SBS. The development of HIVS is one of the most important advances in medicine, showing efficacy and safety, and representing a cornerstone in the management of CIF patients. The development of many surgical techniques and their application in clinical practice to improve their anatomy have contributed to the recovery of intestinal sufficiency and an improved QoL [6,7]. However, the introduction of the semisynthetic glucagon-like peptide-2 (sGLP-2), referred to as teduglutide in medical treatment, has favorably changed the outcomes of these patients, also allowing for the recovery of intestinal autonomy in patients with unfavorable anatomies [8,9,10,11]. In contrast, no important advances have been reported in the medical and surgical treatment of patients with other pathophysiological mechanisms. In selected chronic intestinal pseudo-obstruction (CIPO) patients, palliative surgeries are performed to improve symptoms and QoL, and subtotal enterectomy has been described in severe cases [12,13,14].

**Figure 2:**
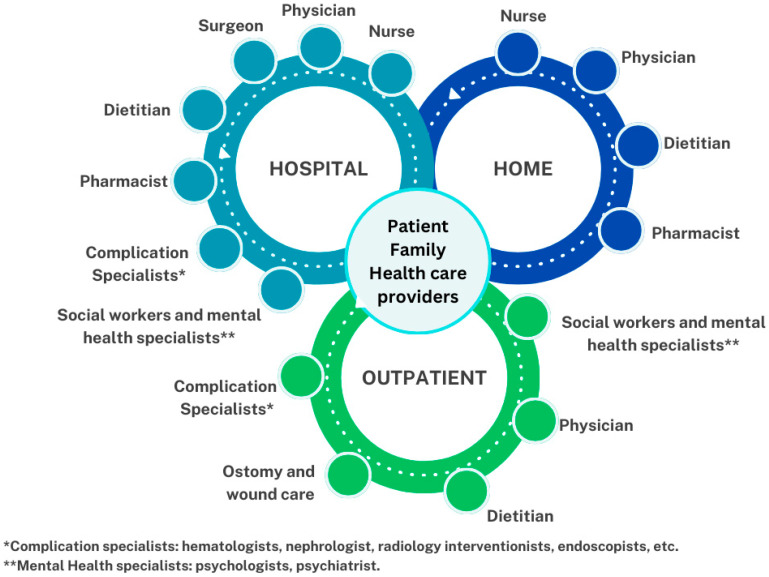
Multi- and interdisciplinary team (MIDT).

In this setting, IF patients are heterogeneous and present with interindividual variations. The best outcomes are achieved if the management is performed by a comprehensive multidisciplinary and interdisciplinary expert team (MIDT) with the capability to offer the following: (a) IVS in acute and chronic conditions; (b) a surgical team with expertise in abdominal wall care, restitution of intestinal transit, and ITx or MVTx; (c) physicians with expertise in the management of CIF and transplant patients, as well as the ability to handle medication on a case-by-case basis and to detect and treat early complications associated with IF, the underlying disease, and/or the IVS; (d) other specialists and allied health professionals who must interact in monitoring both inside and outside the hospital (Figure 2).

However, not all patients around the world have the same access to the resources necessary to treat IF; additionally, in those who do have such access, not all specialized centers use the same treatment criteria.

The aim of this paper is to update the recommendations proposed by expert teams and societies worldwide (ideal management) and compare them with the management carried out in real life (real management) in adult CIF patients.

## 2. CIF’s Current Ideal Management and Real-Life Management

### 2.1. The Disease

#### 2.1.1. Ideal Management

The management of CIF patients should be individualized according to its primary mechanism. In SBS, fistula, and extensive small bowel mucosal disease, malabsorption is the primary mechanism. In CIF-SBS patients, after an enterectomy, their anatomy and physiology change definitively, and the severity of the disease is related to the anatomical type, intestinal length, and absorptive capacity of the remnant intestine. In those with fistulas or SBS with anatomy type 1 (jejunostomy) or 2 (jejunum–colonic anastomosis), the absence of the distal ileum and proximal colon leads to the loss of enterohepatic circulation and peptides (peptide YY (PYY), fibroblast growth factor 19 (FGF19), and glucagon-like peptides 1 and 2 (GLP-1 and GLP-2)). Consequently, gastric emptying and secretions increase, small bowel transit is accelerated, and the bile salt pool is diminished, increasing the risk of gallstones. Complications such as maldigestion, malabsorption and clinical malnourishment, high fistula or ostomy output, dehydration, electrolyte disturbances, acute kidney injury, and small intestinal bacterial overgrowth (SIBO) are the hallmarks. SBS patients with anatomy type 3 (jejunum–ileum–colonic anastomosis) have the most favorable anatomy, and clinical complications could be less severe [15].

In patients with intestinal dysmotility (CIPO) or chronic enteric dysmotility (CED), the primary mechanism is an impairment in gastrointestinal (GI) motility that mimics an intestinal obstruction despite the absence of an obstructive pathology. Clinically, upper GI involvement may cause nausea, vomiting, and consequent malnourishment, while more distal diseases may result in diffuse abdominal pain, abdominal distension, and constipation. In this setting, SIBO occurs and is the cause of malabsorption and secretory diarrhea [16].

**Figure 3:**
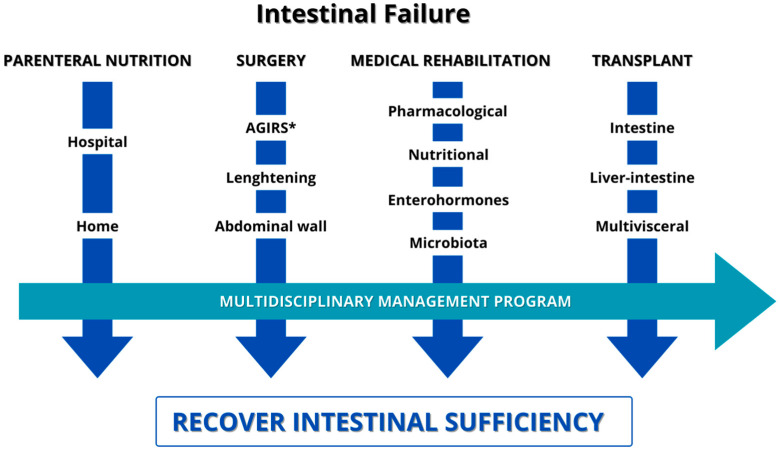
Steps of CIF management (* AGIRS: autologous gastrointestinal reconstruction surgery).

CIF patients are a clinically and metabolically stable population, and their follow-up must be carried out at home because it improves their QoL, decreases intrahospital complications, decreases the health system cost, and allows for the achievement of nutritional, psychological, and physical rehabilitation. All experts agree that HIVS is the cornerstone in the management of these patients, and that surgery and medical treatment are the way to achieve intestinal autonomy in SBS. According to our experience, the treatment of CIF patients should be performed in four steps, implemented in the following order (Figure 3):

First step: HIVS. The aims are to avoid prolonged hospitalizations, improve nutritional and hydration status, maximize patient/family QoL, and minimize the complications associated with long-term support.

Second step: Surgery. All patients should be evaluated by a surgical team to determine whether they are candidates for surgery, with the aim of improving their anatomy and increasing the likelihood of achieving intestinal autonomy and/or improving QoL.

Third step: Medical. Medical management should be carried out before and after surgery, aiming to achieve intestinal rehabilitation either with nutritional support and pharmacological standard medication or by introducing sGLP-2 analogues in selected cases of SBS-CIF. If rehabilitation is not possible, the aim is to improve QoL and reduce complications.

Fourth step: Transplant. Patients who cannot achieve intestinal sufficiency and who develop major HIVS complications should be evaluated for ITx or MVtx.

In recent years, each of these steps has evolved, allowing patients with severe pathologies and unfavorable anatomies to survive and, in many cases, recover intestinal sufficiency and improve their QoL. Ideally, these four steps should be performed by the same MIDT and in the same hospital. This allows for the comprehensive and complete knowledge of the patients and their family, improving the results and the relationship between the patient and the MIDT.

#### 2.1.2. Real-Life Management

Despite the time that has passed since the first description of IF and the advances in its knowledge and treatment, it continues to be an unknown pathology for most physicians and surgeons; it is not taught in many medical schools, and IF and transplant surgeons are not currently recognized as a subspecialty of abdominal surgery. CIF is a very demanding disease for healthcare professionals, the health system, the patients’ families, and the patients themselves, requiring great effort and commitment from all; however, the best results are not always achieved.

The main cause of CIF is SBS, but the underlying diseases vary depending on the continent and the country. Globally speaking, mesenteric ischemia, Crohn’s disease, surgical complications, radiation enteritis, and volvulus are the main etiologies [1]. In Europe and North America (US and Canada), inflammatory bowel disease, surgical complications, and mesenteric ischemia are the most frequent causes [17]. In Latin America, according to the Imp*R*ov*E* Under*ST*anding of Short B*O*wel Synd*R*om*E* registry in Argentina (RESTORE) and the RESTORE amendment that included Argentina, Uruguay, Chile, Peru, Colombia, and Mexico, surgical complications, mesenteric ischemia, and trauma are the most frequent etiologies [18,19]. A report from the Asian continent showed that inflammatory bowel disease, vascular disease, and malignancy were the most common causes of SBS reported in Taiwan [20]. A Chinese study reported intestinal ischemia, inflammatory bowel diseases, malignancy, and radiation enteritis as the main causes [21]. In a Japanese study, Crohn’s disease and volvulus were the main etiologies, and in a Singaporean study, the most common underlying etiologies were malignancy, ischemic bowel, GI dysmotility, surgical complications, and Crohn’s disease [22,23]. The heterogeneity of the disease may indicate different perspectives in the treatment of SBS patients.

Many intestinal fistulas and SBS cases are treated in surgical rooms with the aim of improving abdominal wall damage and local damage without comprehensive management. In many non-specialized centers, there is inadequate knowledge about the pathophysiological changes that occur after an intestinal resection, and patients are discharged from the hospital with a diet or enteral nutrition lacking concomitant medication to control the output or stool movements. This lack of knowledge and expertise leads to the assumption that if the patient “eats”, they are “getting nourished”. In some patients, oral or enteral nutrition produces high stoma or fistula output, or diarrhea, leading to dehydration, electrolyte and trace element disturbances, and malnourishment. The delay in identifying these conditions and the late referral to specialized centers lead to acute kidney injury in patients, as well as the impairment of their clinical condition and outcomes.

On the other hand, the diagnosis of intestinal dysmotility or extensive mucosal disease is difficult. Some patients with CIPO undergo surgery with the diagnosis of mechanical obstruction or are given long-term treatment with diet, prokinetics, or other drugs by poorly trained clinicians and gastroenterologists, delaying the IF diagnosis, worsening the symptoms, and putting patients at risk of malnutrition and impaired outcomes.

### 2.2. The Team

#### 2.2.1. Ideal Management

The successful treatment of CIF patients depends on an individualized approach by a MIDT (seeing at least 20 patients/year) with the ability to understand each pathophysiological mechanism and treat the symptoms of the underlying GI or systemic disease, making appropriate use of the resources [24,25,26]. The aims of a MIDT are to offer comprehensive, safe, and effective support to recover intestinal sufficiency, maximize the remnant intestinal absorptive capacity, minimize the symptoms of malabsorption and the need for IVS, reduce the complications associated with its long-term need, alleviate the burden of daily living, and improve QoL and overall survival. The MIDT should include dietitians with expertise in nutritional assessment and management of oral/enteral nutrition; physicians with experience in IVS and management of pharmacological therapies and medical strategies; surgeons trained in different techniques of abdominal wall surgery, intestinal rehabilitation surgery, and ITx or MVTx; nurses specialized in catheters, ostomies, and wound care; and other specialists, such as endoscopists, radiology interventionists, pathologists, social workers, psychologists, and psychiatrists [27]. The MIDT must have protocols in place for the management of each step of the treatment and should have the capacity to refer patients early to more complex centers, if necessary. Patients, families, caregivers, and healthcare providers are also part of the MIDT, bringing the required commitment and compliance to contribute to the success of the management proposed for each step of the disease (Figure 2).

The evolution of CIF patient care has introduced the need to modify and adapt the constitution of MIDTs based on the evolving clinical condition of the patient, from an inpatient MIDT approach, to an outpatient MIDT follow-up, and then to home MIDT support (patient and family) (Figure 2). Insurance providers and health systems should understand the importance of each component, because the integrated work will improve the outcomes and reduce the number of complications and admissions, as well as the overall healthcare costs.

#### 2.2.2. Real-Life Management

Over time, CIF or ITx teams and their associations have started to modify the treatment scope in practice, towards the constitution of specialized intestinal failure units with the ability to perform an early IF diagnosis and offer the four steps proposed for the treatment in an orderly manner and in accordance with protocol. However, currently, the development of comprehensive MIDTs to treat CIF patients has not been possible in all countries around the world, and in those where such development was possible, the care delivery varies considerably.

There are few physicians specialized in prescribing, detecting early complications related to HIVS, and offering a comprehensive treatment and follow-up of CIF. Trained surgeons are also scarce [28]. In most countries, allied health professionals are not trained in the follow-up of these complex patients; in addition, fewer and fewer physicians and surgeons are interested in treating this type of pathology, because it is very demanding and not very profitable.

Furthermore, the habits and customs of patients, the prevalent CIF etiology, the resources available, and the ability to offer some or all of the proposed steps are different in each country. As a result, the surgical, medical, and nutritional recommendations published in international guidelines cannot always be adapted, making the implementation of protocols tailored to each reality imperative. Unfortunately, most of the centers have not developed such protocols, and many of the decisions in CIF treatment continue to be made by well-meaning but poorly informed physicians or surgeons, leading to wrong decisions and major complications.

According to the ATLAS study, less than 40% of healthcare professionals (HCPs) reported having protocols and national or international guidelines. The main barriers to implementing protocols or guidelines were the lack of training, the need for individualized treatment, and the lack of resources. However, more than 80% reported having protocols and procedures for managing complications associated with CIF [29]. In Latin America, only one center has published its own protocol [30]. In Asia, one center has published its own guidelines for the management of mesenteric ischemia and IF [31].

It is not possible to know exactly how many MIDTs exist worldwide, due to the lack of international registries. In Europe, 74% of HCPs in the ATLAS study reported having MIDTs in their centers. In Latin America, the RESTORE registry and RESTORE amendment showed that not all centers have a MIDT. However, all of them were able to provide IVS and medical management, and some centers were also able to offer surgery, but only a few of them were able to offer the four steps, including ITx and MVTx [18,19].

The shortage of accredited patient referral centers, MIDTs, and protocols, along with the lack of CIF awareness among HCPs, delays diagnosis and early adequate treatment, impairing prognosis and increasing health system costs [29].

The goals of the treatment also vary according to the MIDT. In the ATLAS study, improving/enhancing patient QoL was the most important goal, followed by reducing mortality, achieving intestinal rehabilitation, reducing morbidity, and minimizing adverse events [29]. In the RESTORE registry, the main goals were achieving intestinal sufficiency, minimizing complications, improving QoL, and homogenizing the therapeutic approach [18,19].

To facilitate access to the required therapies, some countries that do not have all four steps make agreements with countries that do. These collaborative agreements are also made between centers in the same country. This organization considers that regional expertise will facilitate treatment for patients from less developed or smaller countries/centers and provide education for physicians. The short-term objective is to resolve the demand for unresolved patients, and the medium- and long-term objective is to train physicians, surgeons, and allied health professionals to complete or create their own MIDTs.

### 2.3. The Therapy (by Steps of Care)

#### 2.3.1. Home Parenteral Nutrition

##### Ideal Management

As patients receive not only parenteral nutrition but also fluid and electrolytes (FEs), the term used in this paper is HIVS. This is a complex, life-saving therapy that has proven to provide effective and safe support for CIF patients; however, it may result in serious harm if not properly prescribed, prepared, and administered. HIVS programs have allowed CIF patients to survive, to improve and maintain their nutritional status, and to live at home, reducing healthcare costs, hospital stay length, and especially decreasing the rate of major complications related to this support, such as intestinal failure-associated liver disease (IFALD), catheter-related bloodstream infections (CRBSIs), and catheter-related venous thrombosis (CRVT), which are indications of ITx or MVTx [32]. HIVS professionals (e.g., physicians, dietitians, nurses) should maintain constant communication with the intestinal failure center as a part of the MITD team (Figure 2).

The criteria for safe HIVS programs are as follows: (1) informed consent signed by the patient and/or the patient’s legal representative must be obtained; (2) patients must be metabolically and emotionally stable; (3) the home environment must be adequate to safely deliver HIVS; (4) patients must have adequate central venous access; permanent catheters are not recommended; (5) patients and caregivers must be able to understand and perform the required procedures for the safe administration of the therapy; (6) patients, family, and/or caregivers should be trained to safely infuse HIVS with appropriate monitoring and early recognition of complications; (7) a formal individualized training program must be provided for patients/caregivers and home care nurses; (8) the prescribed nutrition admixture and ancillaries should be delivered by an experienced/certified healthcare provider; (9) HIVS programs should provide appropriate monitoring and treatment for routine and/or emergency care, with appropriate contact details provided to the patient 24 h per day, seven days per week. The indicators of quality of care are as follows: incidences of CRBSIs, incidences of hospital readmission, and patient QoL [1,32].

An appropriate HIVS admixture prescription should be based on individual patient characteristics and a formal clinical and nutritional assessment. The requirements of fluid and nutrients change during the disease and differ among patients due to the heterogeneity of the pathology (different comorbidities and underlying diseases, anatomy types and intestinal lengths, and ostomy output/stool movements, as well as different nutrition/hydration status and laboratory tests); thus, in the ideal management, HIVS should be individualized.

In order to reduce complications, HIVS must be prescribed and administered by an expert MIDT [1]. To reduce the rate of IFALD, it is recommended to use adequate doses of dextrose to maintain its infusion rate at less than 5 mg/k/minute, along with cyclic administration of HIVS, avoidance of catheter and systemic infections, and the provision of some oral/enteral intakes [33,34]. Additionally, soybean oil lipid emulsion should not exceed 1 gr/k/day. If larger doses are required, lipid emulsions such as olive oil, medium-chain triglycerides (MCTs), and fish oil could be used to reduce the total amount of soybean oil infused. However, there is not strong evidence for the routine use of new lipid emulsions [35,36,37,38].

All international guidelines and experts recommend the use of standardize care protocols to prevent CRBSIs. The British Intestinal Failure Alliance (BIFA) published guidelines aiming to standardize catheter care and reduce CRBSI rates [39]. The appropriate choice of the catheter and the insertion site is essential to reduce the risk of infection. Tunneled single-lumen catheters inserted in upper venous access and avoiding femoral catheters for long-term HIVS are the main recommendations. Peripherally inserted central venous catheters (PICCs) should be considered in some scenarios, such as in tracheostomized patients, or when short-term HIVS is required [40,41]. Management of catheters by highly trained nursing staff, education of patients and caregivers, hand washing, use of 2% chlorhexidine for skin preparation, and full-sterile-barrier precautions are simple measures that have reduced the incidence of CRBSIs [42,43,44]. The use of sterile techniques is mandatory, as it has proven to significantly reduce the rate of infections and, consequently, the risk of developing IFALD. Catheter-locking using an antimicrobial solution has been recommended for the prevention and treatment of CRBSIs. Taurolidine has proven to decrease the occurrence of CRBSIs, and the ESPEN has previously recommended its use for their prevention; however, there is no agreement as to whether it should be used as a primary prophylaxis in all patients or only as treatment for repeated CRBSIs [45,46,47]. Ethanol locks may play a role in preventing CRBSIs for high-risk populations; however, their use has been associated with catheter structural changes, increased rates of catheter obstruction, and systemic toxicity, so their use is not currently recommended [1,48].

Damage to the vein wall during catheter insertion can be minimized using ultrasound-guided catheterization, using tunneled silicon or polyurethane catheters with the smallest diameter, and placing the tip of the catheter near the cavoatrial junction to reduce CRVT. Routine thromboprophylaxis with drugs such as heparin or warfarin is not recommended [49].

The management of HIVS by expert MIDTs has reduced other complications, such as refeeding syndrome, overfeeding, dehydration, overhydration, electrolyte deficiencies, acid–base disturbances, and bone and renal diseases, and has allowed patients to reintegrate into their family, social, and work life [5,50].

##### Real-Life Management

The cornerstone in the management of CIF patients is HIVS. However, in many countries, the development of HIVS programs has not been possible, and in those that do offer this support, the management is not homogeneous. Both the modality of provision and the IVS admixture type differ among and within countries and between benign or malignant CIF.

According to a large international survey that included centers from Europe, the US, Latin America, Asia, and Oceania, the provision of HPN is carried out through two systems: local pharmacies (LPs) and/or home care companies (HCCs). In Denmark, LPs are the exclusive providers, while in the United Kingdom and Israel, HCCs are the exclusive providers. IVS that is premixed, ready to use, and customized to the individual patient’s requirements, with or without additional FEs, constitutes the commercially available admixtures. Premixed, ready-to-use admixtures are prescribed more frequently when LPs are the provider, as well as in cancer patients [51].

In Europe, according to the ATLAS study, of the 12 countries that responded, 87% received routine HIVS, mainly provided by HCCs, and this was more common in Western than in Eastern Europe. In 99% of cases, it was covered by the national healthcare system.

A prospective multicenter observational study from Switzerland showed that cancer was the most common underlying disease, and premixed, ready-to-use admixture was the most common HIVS formula prescribed. Tunneled single-lumen catheters were the most frequently used, followed by port catheters. In 56% of cases, care was provided by the patient themselves or their relatives. The loss of venous access (i.e., catheter displacement and site shift) was the main problem, and the CRBSI rates were lower, with hygiene and aseptic training being the key to reducing infection rates. The use of taurolidine locks was not associated with reduced infection rates [52]. Regarding complications, a Scandinavian study showed that the implementation of simple hygiene insertion bundles reduced CRBSIs [53].

In Latin America, all centers participating in the RESTORE amendment registry were able to provide HIVS through both modalities (HCCs and LPs). All of them had nutritional expert physicians to prescribe and follow the patients; however, not all centers had developed HIVS programs as a part of MIDTs. According to the health policy of each country, the cost is covered by private insurance or the national health system. Tunneled single-lumen catheters of 4–5 French are the most frequently used. In a minority of patients, a PICC is inserted, and no centers reported the use of permanent devices. Additionally, no center reported the use of taurolidine. Argentina is the country with the greatest development in HIVS programs; HCCs and individualized, customized admixtures predominate. In a Brazilian study, the implementation of a bundle and training program effectively reduced CBRSI rates [43]. There are no other publications about HIVS complications.

There are scarce data from Asia, to the best of our knowledge. In a study from Thailand, HIVS was prescribed mainly in oncological patients and was handled by a multidisciplinary hospital nutrition support team. The patients and caregivers received in-hospital training before discharge, emphasizing hand hygiene and aseptic techniques for catheter handling. Tunneled catheters or implanted ports were inserted before hospital discharge, and most patients received HIVS in cyclic infusions [54]. In a Singaporean study, HIVS was administered by patients or their relatives, who were trained at the hospital using stringent catheter-care protocol and guidance from the ESPEN, and their competency was assessed before discharge and at regular intervals during the clinical follow-up. Only a minority had their treatment administered by private nursing staff. Taurolidine was used for those of the patients who had experienced a previous episode of line sepsis. The provider was a LP and delivered the treatment to patients’ homes via courier service. The monitoring was carried out by a nutritional support team [23].

In conclusion, the management of HIVS is not homogeneous, and the modality of provision and the IVS admixture type differ greatly among countries, among centers, and between benign or malignant CIF.

The probability of long-term survival has been published by US and European centers and varies over time (91.8% at 1 year, 69.3% at 5 years, 54.3% at 10 years), as well as being inversely related to the patient’s age [55,56,57,58]. Patients with Crohn’s disease and CIPO showed the highest survival after 5 years (90% and 89%, respectively). Death while on HIVS was reported in 23% of patients, and most deaths occurred within the first 2 years. Underlying GI diseases, complications, or non-gastrointestinal comorbidities were associated with more of the deaths [59]. In those countries in which HIVS is handled by experts, the number of complications associated with its long-term use has decreased, which is reflected in the low numbers of ITxs and MVTxs performed in recent years.

The major problem is the lack of HIVS programs, particularly in low- and middle-income countries. There are few reports in the literature about the management of CIF patients in countries without HIVS. In an Iranian study, the authors performed surgery earlier to restore GI transit or place intestinal valves, with the aim of achieving a specific intestinal diameter in order to perform a lengthening surgery, such as serial transverse enteroplasty (STEP). If these surgeries are successful, enteral nutrition is initiated, with the aim of weaning the patient off hospital IVS; if this fails, early ITx is performed. Long-term outcomes have not been evaluated [60,61].

#### 2.3.2. Surgical Management

##### Ideal Management

Over the years, it has been established that, in patients with enterocutaneous fistulas (ECFs), immediate reoperation can be detrimental, due to the risks of increasing intestinal injury caused by tissue inflammation. Therefore, spontaneous closure should be attempted with adequate medical and nutritional support. Performing a primary reconstruction in a malnourished patient with sepsis, hemodynamic instability, or questionable quality of the remaining intestine (ischemia, inflammatory bowel disease, etc.) is not recommended, and an ostomy should be performed because the anastomosis can fail.

Whenever possible, the distal limb should be exteriorized to enable contrast studies or distal feeding. In these cases, an accurate intraoperative measurement of the proximal intestine must be carried out from the Treitz angle to the ostomy or to the fistula, and distally from the ostomy or fistula to the ileocecal valve (ICV), in order to determine the functional bowel length and the potential absorption capacity of the remnant intestine.

The use of negative-pressure wound therapy has also been standardized, showing no negative impact on mortality or intestinal fistulation.

Recently, it has been proven in Crohn’s disease that up to one-third of ECFs can achieve closure with anti-tumor necrosis factor therapy, and those undergoing surgery within 3 months of infliximab treatment do not suffer from increased complications or morbidity [62,63].

Six months after the procedure to determine the appearance of an ECF, autologous gastrointestinal reconstruction surgery (AGIRS) appears as an innovative and elective procedure to reconstruct or to remodel the intestines [64]. The optimal time is during the chronic phase of IF, when the nutritional status has been optimized, the inflammatory response has resolved, and the general condition (including physical and mental condition) has improved [1,65,66]. The concept of this approach is to use every segment of viable intestine to restore the gut continuity, maximizing the use of the healthy and functional bowel, transforming anatomy type 1 into anatomy type 2 or 3, or modulating the transit time. The re-establishment of the continuity of any segments of bowel (and especially the colon) improves the reabsorption of water and electrolytes. During that time, part of the therapeutic aim is to improve the local condition of the skin, avoiding burn lesions and facilitating re-epithelization.

Changing an unfavorable anatomy to a more favorable one increases the absorptive surface area, allowing for reduced HIVS or achieving intestinal autonomy. Post-surgical anatomy (intestinal length < 75 cm, colon < 57%, absence of ICV) and early citrulline concentration (<20 umol/L) have been described as factors associated with HPN dependence or autonomy recovery, and they should always be recorded and shared with physicians and patients after the procedure in order to assess and plan post-surgical rehabilitation [6,67,68].

Segmental reversal of the small bowel (SRSB) is a procedure that allows transit to be slowed in the absence of bowel dilatation. Surgeons create an antiperistaltic segment of bowel of 10–12 cm in length, which is placed at 10 cm proximal to an end-stoma or small bowel–colon anastomosis. This can be considered to be a complement for AGIRS.

In CIF/SBS patients with anatomy type 2 or 3 and in whom segmental bowel dilatation with poor peristalsis develops, small intestinal bacterial overgrowth (SIBO) causes malabsorption and could be the reason for not achieving intestinal sufficiency or for needing to restart HIVS in patients who were previously able to wean off this support. If medical treatment of SIBO is not effective, longitudinal intestinal lengthening and tailoring (LILT; described in 1980) or serial transverse enteroplasty (STEP; described in 2003) should be considered in highly selected stable adult patients [69,70]. Both procedures increase gut adaptation and reduce the risk of SIBO. STEP has also been applied to the colon, where it is called serial transverse coloplasty (STCP), and used in SBS to reduce colonic bacterial overgrowth [7]. Major complications associated with lengthening procedures can develop. The detriment of the normal architecture of the intestinal intrinsic nervous system and the muscle layers that allow for coordinated motility should make these procedures less attractive to consider. Therefore, in this case, increasing the length of a segment of intestine does not increase the absorptive capacity.

In CIF, secondary to intestinal dysmotility, extensive mucosal disease, and mechanical obstruction, the aim of surgery is to improve the symptoms (gastrostomy, ileostomy, or both). Therefore, the surgical decision should be made by experienced surgeons, taking into account the pathophysiological mechanism, etiology, and patients’ clinical and nutritional conditions [71]. These procedures have been shown to improve QoL and oral intake, and to reduce the need for suction gastrotomy and hospital admissions [12,13].

In some cases, the subtotal enterectomy should be considered for severe CIPO cases that are refractory to medical treatment [14].

The recently described Trifecta procedure (subtotal colectomy, pyloroplasty, and chimney ileostomy) is another surgical option to enhance oral tolerance, modify transit time, ameliorate SIBO, and serve as a bridge to transplantation in patients with severe intestinal dysmotility [7].

In chronic radiation enteritis, the final fibrosis and vasculitis cause narrowing in the intestinal loops, with dilation of the bowel proximal to the stricture, thickening the affected segments of the intestine and serosa. Consequently, severe stenosis, ulceration, necrosis, and perforation of the intestinal wall can occur, requiring ostomies and/or extensive enterectomies that can result in SBS. In this setting, the CIF is due to two pathophysiological mechanisms, i.e., extensive mucosal disease and the need to complete a bowel resection leading to SBS [4,12,71,72,73].

##### Real-Life Management

Despite the extensive communication of the concepts described above, post-surgical complications, mostly as a consequence of primarily trying to close intestinal perforations or failed anastomosis, have become the primary cause of CIF-SBS in adult patients.

In 2019, a major experience in the surgical management of CIF was published. A total of 790 (1.9/patient) reconstructive/remodeling procedures were performed. The most frequent reconstructive procedure was autologous gut reconstruction, which recovered nutritional autonomy in 71% of patients. The lengthening of the small bowel and colon (STEPS/STCP) was the second-most frequently used procedure. STEP was performed concomitantly with autologous gut reconstructions in most cases, and intestinal autonomy was achieved in 72% of cases. Trifecta was performed in a selected group of intestinal dysmotility patients, achieving a higher rate of nutritional autonomy compared with single or combined reductive/decompressive interventions. The interposition of visceral conduits (colonic and jejunal) to restore the alimentary flow was also performed. Colonic conduits were used to restore the flow between the cervical esophagus and the stomach or jejunum in patients with congenital or acquired esophageal pathology, or to repair major duodenal defects in SBS patients with Crohn’s disease. The jejunal conduits re-established alimentary flow in patients with resected stomachs and SBS, with the creation of a neo-stomach [7]. The most frequent surgical complications reported were anastomotic leak/bowel perforation (most due to thermal injuries), postoperative bleeding, and infections. Only 10% of patients died, due to surgical failure. The survival rates at 1 and 5 years were 88% and 74%, respectively.

**Figure 4:**
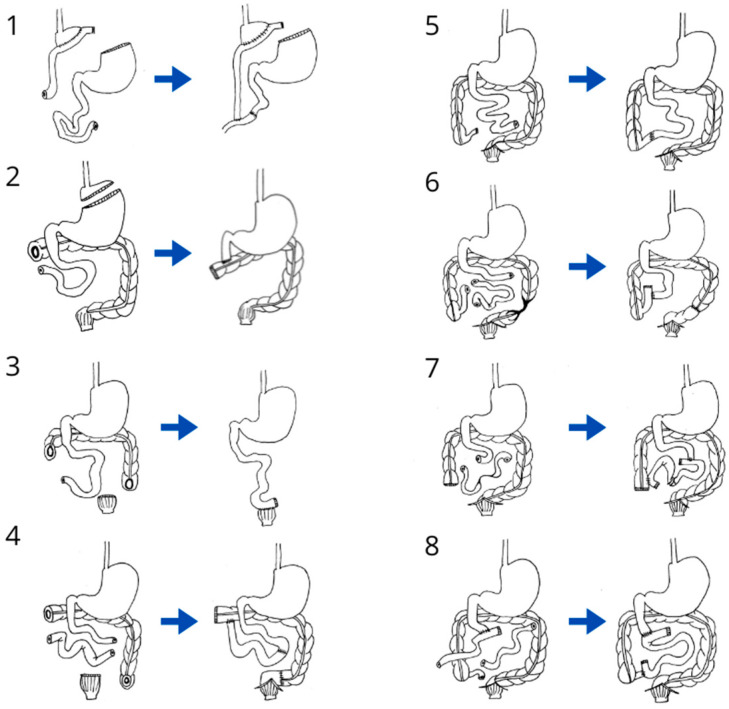
Examples of different autologous GI reconstruction surgeries (AGIRSs) that need to be adjusted to each individual case in order to obtain a more favorable final anatomy type. (1) and (2): Examples of AGIRS after bariatric surgery. (3): Example of an AGIRS requiring resection of remnant colon and ileorectal anastomosis. (4): Example of an AGIRS with 2 anastomoses and rescuing an abandoned intestinal segment. (5): Example of an AGIRS performed close to the ICV to obtain a type 3 anatomy. (6): Example of an AGIRS that requires sacrificing an abandoned segment, involved in a complex enterocutaneous fistula, with simultaneous removal of a dominant colonic stricture. (7): Example of an AGIRS requiring removal of a segment of small bowel stricture. (8): Example of an AGIRS requiring removal of an injured proximal intestinal segment and its replacement with a more preserved distal and abandoned one, obtaining a type 3 anatomy.

A major experience in the management of adult CIF patients in Latin America was also published in 2019. A retrospective analysis was conducted in 88 patients. In all patients, AGIRS was performed and improved the initial unfavorable anatomy, converting anatomy type 1 to type 3 in 75 out of 78 patients. The surgical techniques are summarized in Figure 4. The mean number of anastomoses performed was 1.4 per patient. The mean post-surgical intestinal length was 159 ± 103.4 cm. The most frequent postoperative complications were wound infections, intra-abdominal bleeding and intra-abdominal collections. The mean postoperative time to achieve intestinal sufficiency was 817 ± 842 days. A formula was developed to predict PN-free survival with logistic regression. The variables included were intestinal length, presence or absence of ICV, and use of enterohormones (EHs) [6]. Surgical management of CIF patients has become the cornerstone of the treatment of SBS and fistulas, allowing for improvements in the anatomy and increasing the possibilities for achieving intestinal sufficiency with medical and nutritional management [74].

In CIPO patients, the partial or extended surgical resection of the dilated small bowel can improve symptoms and oral intake [74]. According to a French study, surgery was required in 84% of CIPO cases, with 3 ± 3.3 procedures per patient, and 37% experienced surgery during the first symptom occurrence before CIPO diagnosis. After diagnosis, the surgical procedures (laparotomy, intestinal resection, and venting or ending ostomy) decreased from 2.2 ± 5.3 to 0.3 ± 0.5. SBS was the result of bowel resection in 37% of cases, with a mean remnant bowel length of 68 ± 56 cm, and two patients underwent ITx [75,76]. Associated SBS did not worsen the survival of adult CIPO patients.

#### 2.3.3. Medical and Nutritional Management

##### SBS and Fistula Patients

Ideal Medical Management

High ostomy output and diarrhea often pose a therapeutic challenge; hence, individualized therapy including dietary and oral fluid modifications, along with antimotility and antisecretory drugs, is the first choice for treatment [49].

Antimotility drugs:

- Loperamide and diphenoxylate/atropine are the first-line antimotility agents due to their low incidence of systemic side effects, and they should be administered 30 min before meals and bedtime [77]. Loperamide’s normal uptake is in the enterohepatic circulation, so patients with anatomy types 1 and 2 require higher doses than conventionally recommended (up 32 mg/day) to achieve a therapeutic response [78].
- Codeine and tinctures of opium are second-choice drugs and are used in the treatment of refractory diarrhea. The metabolism of opioids varies according to age and whether the patient is a poor or ultrarapid metabolizer; therefore, the antidiarrheal regimen must be individualized, taking these factors and other comorbidities into consideration [79].
- Clonidine, an agonist of ∝_2-_adrenergic receptors in the enterocytes, is effective for the treatment of chronic diarrhea resulting from longstanding diabetes [80,81]. Animal and human studies have shown that a dose of 0.3 mg/day has significant antimotility effects and prolongs the intestinal transit time, increasing nutrient and fluid absorption, while decreasing fecal sodium and potassium losses. Transdermal administration has been associated with a modest but clinically significant decrease in fecal ostomy output in SBS patients [82,83,84].

Antisecretory medication:

- Histamine 2 receptor antagonists (H2RAs) and proton-pump inhibitors (PPIs) reduce the volume of gastric secretions during the period of greatest hypersecretion (6–12 months after intestinal resection). It is worth noting that H2RA therapy can be added to HIVS for easy administration to decrease gastrointestinal secretions as a response to the nutrients being delivered.
- Somatostatin and somatostatin analogues such as octreotide inhibit secretagogue-induced water and electrolyte secretion in the jejunum and the colon, stimulate sodium and chloride absorption in the ileum, and inhibit the release of various GI and pancreatic hormones that may contribute to diarrhea (e.g., VIP, GIP, gastrin), decreasing gastric, biliary, and pancreatic secretions and, consequently, the volume of ostomy output. They improve blood circulation in the intestinal wall, reduce absorption of bacterial toxins, accelerate the resolution of inflammation, and stimulate T-cell proliferation [84]. Conflicting results have been reported, and high-quality evidence supporting their clinical benefit in SBS is lacking, so their use should be evaluated in each case [84,85,86,87] and considered when the first-line treatment drugs are ineffective [88,89]
- Bile acid sequestrants (cholestyramine) should be considered in anatomy type 3 if diarrhea is caused by the colonic toxicity of poorly absorbed bile salts. In anatomy type 1 or 2, the bile acid pool is reduced, so their use is not recommended, because steatorrhea and malabsorption of fat-soluble vitamins can worsen.
- There is no evidence of pancreatic exocrine deficiency in SBS, so the use of pancreatic enzymes should be considered in patients with accelerated intestinal transit and poor mixing of nutrients and enzymes due to asynchrony of pancreatic juice secretion. In this setting, their prescription must be evaluated in each case.

Table 1 shows the recommended doses of each medication.

After a surgical procedure that improves the anatomy, most SBS/fistula patients recover intestinal sufficiency with a correct use of these drugs together with an adequate nutritional strategy. For those who remain dependent on HIVS, the use of the semisynthetic glucagon-like peptide-2 (sGLP-2) should be considered.

Native glucagon-like peptide-2 (nGLP2) is a 33-amino-acid peptide secreted by intestinal L cells of the distal ileum and proximal colon in response to the presence of nutrients in the gut lumen; it promotes intestinal crypt-cell proliferation, increases villus height and crypt depth, inhibits enterocyte apoptosis and gastric acid secretion, decreases small intestinal motility, and increases mesenteric blood flow. It is degraded by dipeptidyl peptidase IV (DPP-IV), and its mean elimination half-life is 7 min.

The sGLP-2s are a novel group of drugs that have changed the course of SBS-CIF treatment.

- Teduglutide (TED) results from the substitution of one amino acid in nGLP-2, making it resistant to degradation by DPP-IV, thereby increasing its half-life to 1.3 h in SBS. TED is absorbed rapidly, achieving maximum plasma levels 3–5 h after dosing; its clinical safety and efficacy for a subcutaneous dose of 0.05 mg/kg/day, or a 50% reduction in the dose in renal failure patients, were demonstrated in the phase III Study of Teduglutide Effectiveness in Parenteral Nutrition Dependent Short Bowel Syndrome Subjects (STEPS), carried out in centers in Europe and the US [8]. The extension trials, STEPS-2 and STEPS-3 (the latter conducted only in the US), along with other studies, showed consistent safety and efficacy with a progressive reduction in HIVS over time [9,10]. In Japan, the short- and long-term efficacy, safety, and pharmacokinetics of TED were analyzed in adult and pediatric Japanese patients with SBS-CIF. There were no differences in the maximum concentrations of TED between this population and non-Japanese patients, the treatment was associated with clinically meaningful reductions in HIVS requirements, and no new safety concerns were identified for this population [11,22,91]. With all of this evidence, the ESPEN recommended that TED should be the first choice for a carefully selected group of SBS-CIF patients dependent on HIVS, but it should be prescribed by an expert team capable of following these patients [46]. Before starting the treatment, complete laboratory tests, ruling out pregnancy in fertile women, and a colonoscopy and gastroscopy to rule out or remove polyps due to their potential to cause hyperplastic changes, are recommended by all experts. Subsequent colonoscopies should be carried out according to guidelines for surveillance after polypectomy, but no less frequently than every 5 years. Other imaging tests should be evaluated on a case-by-case basis. All patients must be monitored closely, especially those with predictors of early response, because rapid changes in fluid balance (even during the first 4 weeks) can cause fluid overload or increase the absorption of medication, indicating that a reduction in HIVS volume and/or medication dose is needed [92]. To adjust the volume, the algorithm proposed in STEPS can be used in clinical practice. The reduction in energy requirements should be determined by estimating energy balance using measures of changes in food intake, fluid balance, and body composition. The frequency of monitoring may be adapted based on an individual’s adverse effects, and assessment should be performed consistently by the expert treating physician.

In recent years, other molecules have been developed:

- Apraglutide results from the substitution of four amino acids in nGLP-2, with a longer half-life of 72 h due to low clearance resulting from DPP-IV resistance and high protein binding, making it suitable for once-weekly dosing. In a phase II trial, two doses (5 and 10 mg) were tested, both of which were well tolerated and significantly decreased the volume output [93]. Currently, a phase III study (STAR) is ongoing.
- Glepaglutide differs from nGLP-2 in the substitution of nine amino acids and a C-terminal tail with six lysin residues; its half-life is 50 h, and it is administered once or twice weekly. Phase II studies have been published; however, the results of the Efficacy and Safety Evaluation (EASE) phase III clinical trials are not yet available [94].

The main aim of medical and nutritional treatment in SBS/fistula patients is to recover intestinal sufficiency and improve QoL. The management of MIDTs and the introduction of sGLP-2 have favorably changed the outcomes of the disease, allowing patients with unfavorable anatomy to be weaned off HIVS.

Ideal Nutritional Management

The nutritional management should be individualized; thus, before its implementation, a complete nutritional assessment must be performed. However, there are no specific tools designed for nutritional evaluation in this population. The following parameters may be included:

- Weight: Unintentional weight loss could indicate malnutrition. However, it could be influenced by hydration status.
- CT scan: Muscle mass at the third lumbar vertebra correlates significantly with whole-body muscle mass. Combined with strength assessments (e.g., handgrip strength), this allows for a diagnosis of sarcopenia.
- Bone density: This helps determine the presence of osteopenia or osteoporosis.
- Laboratory workup: Serum levels of electrolytes, vitamins (e.g., B12, folic acid, D), trace elements (Cu, Zn, Se, and Fe), and C-reactive protein (CRP) should be included in the analysis. It is important to consider that some of these parameters must be correlated with inflammatory status.
- Urine output: This is useful for evaluating hydration status and should be >0.5 mL/k/h or >1 mL/k/h in patients with kidney stones or renal failure. Determining 24 h urine urea nitrogen allows for achieving nitrogen balance and assessing adequate protein requirements.
- Stool/ostomy output: Some nutritional modifications must be performed to achieve an ostomy output of less than 1000 mL/day, or to decrease the number of stool movements.
- Physical exam: Examination of skin, hair, nails, eyes, etc., is helpful to detect suspected micronutrient deficiencies [95].
- Dietary records are useful to assess oral intake and allow for identifying and correcting some dietary habits, educating the patients, and tailoring an individualized diet.

Once the assessment is complete and a nutritional diagnosis is possible, a nutritional strategy can be implemented. It is known that the main stimulus to promote intestinal adaptation (a natural process characterized by structural and functional changes in the remnant small intestine and, to a lesser degree, in the colon) is the presence of polymeric nutrients in the intestinal lumen. These nutrients stimulate crypt-cell hyperplasia, enteric hormonal production, growth of villi, and intestinal secretions [96,97]. The presence of the ileum and proximal colon (anatomy type 3) ensures the secretion of native enterohormones, the enterohepatic circulation, the absorption of fluid and electrolytes, and the recovery of energy through the presence of microbiota. This process can last up to five years. The energy requirements may be established using indirect calorimetry; otherwise, predictive formulas could be used [98].

Dietary recommendations differ according to the anatomy type (Table 2). The role of fiber is controversial, though it can be effective for some groups of patients and with some types of soluble fiber; its use can be limited by side effects, including bloating and flatulence.

To maintain hydration, oral rehydration solutions are recommended, particularly in those with end-jejunostomy, because the Na+-coupled glucose transport system resides mainly in the jejunum.

Each nutritional strategy should be monitored and corrected if necessary. Continuous follow-up by the MIDT is mandatory, even for those patients who have been weaned off HIVS, because some of them can evolve to intestinal insufficiency, requiring oral nutritional supplementation, oral rehydration solution, or enteral nutrition.

Real-Life Medical and Nutritional Management

Although the ESPEN has published recommendations for the management of CIF patients, the pharmacological and nutritional treatment in real life varies among countries and centers [1]. The pharmacological variations are related to the team’s expertise and the access to medication. Variations in dietary prescription are influenced by patients’ nutritional habits and preferences, making the intervention of an expert dietitian in the MIDT essential to evaluate each case.

Real-Life Medical Management

According to a European survey, IF centers uniformly use acid suppression, but other approaches differ significantly: 82% use opioids (mainly loperamide), 78% use octreotide, and 65% use an oral rehydration solution [99]. The use of loperamide and codeine together was reported to be effective in the treatment of diarrhea in SBS/fistula patients [86].

In the North America–Europe SBS Management Working Group, loperamide was reported as the first-line antimotility medication and the most frequently prescribed, using doses of up to 64 mg daily. On the other hand, diphenoxylate/atropine was not used in any center in this study. The first-line and most frequent antisecretory medications reported were PPIs. H2RA and octreotide were used in only 10% of centers. It is not clear how long antisecretory medication should be administrated after surgery, but most of the centers continued indefinitely, and only a minority stopped the treatment after 6 months. Bile acid binders were used in 30% of colon-in-continuity (CiC) patients. Pancreatic enzymes and probiotics were used occasionally, and the use of clonidine was not reported [97]. In adult patients, spontaneous adaptation, surgical procedures to improve the anatomy, diet counselling, and antisecretory and antimotility drugs have allowed CIF-SBS patients to be weaned off HIVS in 20% with anatomy type 1 and more than 100 cm of small bowel, 40% with anatomy type 2 and more than 65 cm, and 80% with anatomy type 3 and more than 30 cm of small bowel [67,68].

There have been no data published by specialized centers from Asia and Oceania about their protocols for medical treatment in CIF-SBS/fistula patients. According to the RESTORE amendment and a review of middle-income countries, most specialized centers follow the ESPEN recommendations [19,100]. In our center, loperamide and PPIs are the first-line antisecretory and antimotility drugs, respectively, and in those patients in whom PPIs are not effective or who cannot continue with IV administration, H2RA is added to the HIVS formula. In most patients, antisecretory medication is stopped after 6 months of treatment. In those patients with high ostomy output who behave as net secretors, octreotide is prescribed early in the treatment. Clonidine, antibiotics, or pancreatic enzymes are used on a case-by-case basis. With this pharmacological management after surgery and an adequate diet, 68.5% of patients with SBS-CIF recovered intestinal sufficiency in our center [30]. Other centers in Latin America have published medical recommendations based on international studies [101,102].

Those patients who remain dependent on HIVS should be evaluated to receive sGLP-2. Currently, TED is the only sGLP-2 commercially available; it is approved for the treatment of CIF-SBS in a selected group of patients. The criteria for eligibility are not homogeneous in all specialized centers, having even been prescribed in patients with fistulas, ITx, and cancer, so the comparison of results among programs is difficult [103,104].

The ESPEN uses two criteria: (1) the time elapsed since the last intestinal resection (12–24 months to ensure intestinal adaptation), and (2) the absence of contraindications (published in STEPS) [1]. In a cross-sectional observational study in a single center in Italy, the candidacy was categorized into three groups: (1) non-candidates (34.2%) with contraindications, (2) potential candidates (30.4%) with comorbid conditions that require special precautions, and (3) straight candidates (35.4%) with no contraindications or specific comorbidities [105]. In a retrospective observational study in the United Kingdom, 48% of patients with CIF-SBS were deemed suitable for TED treatment, with comorbidities being the most frequent cause of unsuitability [106]. In a single-center study from France, the inclusion criteria were remnant small bowel length < 250 cm and HIVS dependence. The ineligibility criteria were cancer, spontaneous weaning, and the presence of comorbidities. This team suggested the early use of TED [107]. In a recent paper, most experts recommended its use in stable patients, usually 12 months after the intestinal resection (range: 6–36 months), with optimized and stabilized HIVS. Eligibility is assessed by reviewing the patient’s medical history, individual needs, clinical and nutritional status, and adherence to treatment [92].

In a retrospective analysis of a single center in the US, TED was started in CIF-SBS patients following a period of stabilization to ensure adequate nutrition and hydration status as well as patient compliance with therapy [108]. According to a large commercial database in the US, TED was prescribed to 170 patients between 2015 and 2019, including ITx patients and those with prior GI malignancy and GI neoplasm [104].

In Latin America, our center has published criteria for candidacy for TED treatment [109]. According to our protocol, CIF-SBS patients are candidates if (a) they have a benign etiology; (b) after surgery to improve the anatomy, the patient is left with SBS and is dependent on HIVS; (c) there are no contraindications, including inadequate adherence to treatment; and (d) there is insurance to cover the cost.

In those candidates, the initiation of the treatment is divided into two groups: (1) those who, despite surgery, an adequate diet, and medication, are unable to continue decreasing their HIVS volume over a period of 6 months and, after ruling out secondary causes of malabsorption, are started on TED; and (2) those who develop major HIVS complications, in which case the treatment is started immediately after surgery to avoid ITx. The patient, family, and caregivers are informed about risks, benefits, and adverse events; they are trained in the subcutaneous injection technique and should be willing to comply with the therapy, including daily self-administered dosage [30].

In real life, the overall response rate during TED treatment was estimated between 64% at 6 months and 82% at ≥2 years [110]. Patients with the highest baseline HIVS volume requirements had the greatest response at week 24, and predictors associated with an early TED response included the presence of stoma, as well as the absence of colon-in-continuity and inflammatory bowel disease [111,112]. The weaning-off rate was estimated to be 11% at 6 months and 21% at ≥2 years, and the presence of colon-in-continuity was associated with an increased weaning rate. Slightly better outcomes, without significant differences among patients with SBS due to Crohn’s disease, could be observed in both the response and weaning rates [110]. Data from the ongoing, prospective, observational, multinational SBS registry demonstrate that TED has clinical benefits, in a real-world setting, upon up to 4 years of treatment in slow responders [113]. Similar results were observed in a Japanese study with the longest evaluation period ever reported (4.5 years) [11]. Table 3 shows the results of TED treatment in some specialized centers.

The most frequently reported adverse effect was abdominal pain, and the serious adverse effects reported included CRBSI, headache, nausea, and intestinal obstruction, among others. Most real-life programs reported that laboratory tests and colonoscopies were performed before starting treatment. However, according to a US database, 47% of patients received the treatment without a colonoscopy [104]. No malignancies related to treatment were reported, but 12% and 18% of patients presented colonic polyps before and after the treatment, respectively [114]. The presence of multiple new duodenal hyperplastic polyps was reported in a patient after 26 months of treatment, and 10 out of 35 (28.6%) patients treated in a single center for >1 year developed polyps in their duodenum and colon [115,116].

In real life, there is no consensus on how to decrease the volume and calories during TED treatment. According to published data, TED discontinuation was due to adverse events, GI symptoms, lack of significant benefit, diagnosis of non-GI cancer, injection site pain, patients’ decisions, and poor compliance [107]. In our program, some patients were able to suspend HIVS and TED treatment due to pregnancy, weight gain, abdominal distension, or constipation; none of these patients required HIVS restoration. Other causes of discontinuation were poor adherence, lack of significant benefit, lack of coverage, and the death of two patients during the SARS-CoV-2 pandemic [30].

**Table 3:** Results of TED treatment in some specialized centers.

Author (year)	Country	N	SBS Etiology (%)	Anatomy Type: *n* (%)	Intestinal Length cm: Median (Range)	Time in TED (Range)	Weaning (%)	Response/No Response	Protocol
Lam, K. et al. (2017) [107]	USA	18	CD MI Others	1:4 2:9 3:5	55 (15–180)		11/18 (61)	78%/11%	Yes
Puello, F. et al. (2020) [117]	USA	18	CD MI Others	1: 10 (55.6) 2: 3: 3 (16)	100 (40–240)	3.2 years (0.6–6.2)	5/18 (27.7)	100%	No
Harpain, F. et al. (2021) [118]	Austria	13	CD (61.5) SC (23.1) MI (15.4)	1: 3 (23.1) 2: 7 (53.8) 3: 3 (23.1)	106.7 ± 25.2	107 weeks (26–205)	12/13 (92)	100%	No
Joly, F. et al. (2019) [119]	France	54	MI (39) CD (30) VO (7.13)	1: 19 (35) 2: 33 (61) 3: 2 (4)	61.8 ± 5.9	24 weeks	13/54 (24)	85%/15%	No
Pevny, S. et al. (2019) [120]	Germany	27	MI (44) IBD (15) SC (15)	1: 6 (22) 2–3: 21(78)	205 ± 173 45 ± 34	104 weeks	4/19 (21)	79%	No
Schoeler. M. et al. (2018) [121]	Germany	14	CD (50) MI (36)	1: 5 (36) 2–3: 9 (64)	64.5 ± 43.3	15.7 ± 7.05 months	2/14 (14.3)	85.7%	No
Solar, H. et al. (2020) [108]	Argentina	17	MI (47) SC (17) VO (17)	1: 1 (5.9) 2: 9 (53) 3: 7 (41)	37.9± 28.7	116 weeks ± 69.1	8/12 (66.6)	94%/6%	Yes
Nakamura, S. et al. (2022) [22]	Japan	18	CD (78) VO (17) Other (5)	1: 11 (61) 2–3: 7 (39)	219.42 ± 147.15	24 weeks		50%	

CD: Crohn’s disease; MI: mesenteric ischemia; SC: surgical complications; VO: volvulus; IBD: inflammatory bowel disease.

Real-Life Nutritional Management

Data published by the North America–Europe SBS Management Working Group show variation regarding dietary recommendations due to cultural differences, highlighting the need for individualized dietary education [103]. Diet should be tailored to each patient based on regional, cultural, and personal dietary patterns, with the goal of patients understanding the relationship between their therapeutic diet and optimization of health and hydration. For those without CiC, dietary prescriptions were consistent with expert recommendations and ESPEN guidelines [1,96,98]. In contrast, not all recommendations were followed in patients with CiC; a high-sodium diet was not frequently recommended (clinicians felt the absorptive capacity of the colon rendered this recommendation less critical), and a low-fat diet was prescribed in only 35% of patients. This discrepancy might represent a knowledge gap or differing perspectives on what constitutes a low-fat diet, or it could represent the practical experience that does not provide an overall benefit in the short term [122]. Additionally, a low-fat diet reduces overall intake, given that it is often less palatable and provides lower levels of essential fatty acids and fat-soluble vitamins [1]. Soluble fiber was used in some patients with CiC to promote its fermentation into short-chain fatty acids (SCFAs) by the colonic microbiota.

The restriction of oral hyperosmolar fluid is recommended by all centers and is strongly recommended for patients without CiC. Hypo-osmolar fluids and sports drinks were used in 50% of centers. Homemade oral rehydration solutions were more frequently used than commercial ones [122].

In Latin America and the Caribbean, there are no published data on the nutritional management of oral/enteral CIF-SBS/fistula. In our center, all diets and hydration are individualized and prescribed by an expert dietitian, following the published recommendations of the ESPEN. These plans take into account individual preferences, nutritional habits, remnant intestinal anatomy, underlying disease, nutritional assessment, ostomy output or number of stool movements, and stool characteristics (we use the Bristol scale as a reference). In patients with CiC, the restriction of fat is intended to avoid the formation of oxalate stones, and in patients without an ileum it is due to the lack of absorption. Soluble fiber is used in CiC to promote SCFA production; however, individual tolerance is prioritized. In our center, commercial oral rehydration solutions are the most frequently used, and we do not use sports drinks. The dietary and fluid intake is agreed upon with a physician, who must adjust the medication to increase its absorption. We modify the diet if (a) ostomy output is more than 1000 mL per day or the number of stool movements is more than six per day (or less than six but impairing QoL); (b) the Bristol score is 6–7; or (c) the patient is dependent on HIVS to maintain renal function and electrolyte balance due to high ostomy output or diarrhea, despite having adequate medication.

##### Non-SBS and Non-Fistula Patients

Ideal Medical Management

The aims of medical treatment in patients with chronic intestinal dysmotility, extensive mucosal disease, and mechanical obstruction vary on a case-by-case basis. However, most of these patients will not recover intestinal sufficiency due to the chronicity and irreversibility of the underlying disease and the lack of specific treatment, requiring long-term or lifelong HIVS.

In CIPO/CED, the objectives of the treatment are to reduce symptoms, improve intestinal propulsion, maintain adequate nutritional and hydration status, avoid vitamin and trace element deficiencies, and reduce the major complications related to long-term HIVS requirements. Abdominal pain is a common symptom and has multiple mechanisms, making its treatment difficult. Opioids exacerbate intestinal dysmotility and are associated with SIBO, dependence, and risk of CRBSIs [123]. The absence of a phase III migrating motor complex (MMC) is correlated with bacterial translocation and can result in potentially life-threatening consequences [123]. Unfortunately, there are no motility agents that are able to restore normal GI motor function. Most prokinetics (e.g., metoclopramide, domperidone, prucalopride, erythromycin, amoxicillin/clavulanate, octreotide, and neostigmine) are helpful only in a minority of patients [124]. Subcutaneous octreotide induces phase III MMC in the small intestine in CIPO secondary to scleroderma [125]. The role of the gut–brain axis and neuromodulation in abdominal pain has allowed the use of tricyclic antidepressants as well as selective serotonin or serotonin–norepinephrine reuptake inhibitors. Gabapentin or pregabalin can be helpful for neuropathic pain [124,126]. The reduction in opioid therapy and the use of antibiotics are the most efficient strategies to avoid dysmotility exacerbations and SIBO. SIBO treatment reduces malabsorption and improves nutritional status and bloating, with metronidazole, rifaximin, tetracycline, amoxicillin/clavulanate, doxycycline, and norfloxacin being the most useful antibiotics (Table 1).

In chronic radiation enteritis patients, SIBO and/or pancreatic insufficiency should be treated to improve symptoms and malnutrition. The medical treatment includes corticoids, pentoxifylline, and hyperbaric oxygen; however, there is no strong evidence that these therapies prevent the progression of this pathology. HIVS and bowel rest for some months can achieve a spontaneous resolution of intestinal obstruction, allowing for the resumption of oral feeding without surgical intervention [127,128].

Ideal Nutritional Management

According to the published recommendations, patients with adequate intestinal absorption should be encouraged to take small, frequent liquid meals (5–6 per day) while avoiding high-fat, high-residue (delaying gastric emptying), and high-lactose/fructose (evoking bloating/discomfort and SIBO) foods. However, most of these patients present discomfort independent of the meal served, and their tolerance is influenced by the extent of the disease [1]. Micronutrients should be monitored, and vitamins A, D, E, and K, as well as B12 and folic acid, should be supplemented when needed. Up to two-thirds of patients with CIPO develop nutritional problems or specific nutrient deficiencies. In those patients with IF, HIVS should be implemented in specialized referral centers [124]. MCT can also be used [129,130]. In cases of inadequate oral intake, enteral nutrition with standard, non-elemental formula should be considered. When delayed gastric emptying is present, bypassing the stomach and directing the feeding into the small intestine is recommended, starting with a slow infusion, and continuous feeding or cyclical feeding (overnight) is preferred to large bolus feeding [131]. All of these patients must be evaluated by an expert dietitian.

Real-Life Medical Management

The treatment of CIPO patients is difficult and should include psychological and clinical approaches. In a UK study, the use of 1.5–2.0 g/day of oral erythromycin (a motilin analogue) was found to be helpful in 6 out of 15 (40%) CIPO patients, relieving obstructive episodes and symptoms [132]. In a French report, during and between CIPO exacerbations, all patients received propulsive therapy (metoclopramide, domperidone, octreotide), with incomplete or transient efficacy. Requirements of at least two prokinetics in combination with PPIs, as antisecretory drugs and sequential antibiotic therapy for SIBO treatment, were systematic. Laxatives and/or enemas were prescribed in 57% of patients. Analgesics and/or antispasmodics were prescribed in all cases requiring intermittent or permanent narcotic medications [76]. In an international survey, most of the clinicians reported that factors such as bacterial overgrowth, nutritional impairment, and pain are relatively easy to treat, but that there are no proven therapies that restore gut motor function. According to this study, domperidone, metoclopramide, erythromycin, and prucalopride were the most frequent drugs used in CIPO and CED [133]. The evidence demonstrates that there are few reports on the management of CIPO and CED patients, with wide variations in practice.

Real-Life Nutritional Management

In real life, there are few reports on the nutritional management of CIPO patients. In a French study, a decrease in patients’ BMI from the first symptoms’ occurrences to the time of diagnosis was reported. The delay between the first symptoms’ occurrences and HIVS initiation was 3.2 (0–23) years, and the duration of this support was 2.5 (0–21) years, with a dependence degree of 64 ± 25%. An important improvement in BMI was observed with diet modifications and HIVS management. Considering the oral intake, 33% had adaptive hyperphagia, whereas 30% presented total oral intolerance. The dietary protocol published consisted of low-residue, low-fiber, and continental lifestyle meals. In cases of significant vomiting, a bowel rest period of 3–4 weeks was advised before restarting feeding. Solid food was authorized in case of the absence of poor oral tolerance, significant nausea, or vomiting. Oral intake (i.e., total energy, fat, and carbohydrates) was evaluated by a trained dietitian during a stable 3-day period, and it was a major independent factor associated with better survival and lower HIVS dependence. However, treatments to enhance oral feeding capacity are lacking, as prokinetic drugs only provide manometric benefits and only slightly improve clinical status. HIVS was delivered through either a central tunneled venous catheter or tunneled port-a-cath. Cyclic nocturnal parenteral nutrition infusion was performed at home in all patients, either by the patients themselves or under nurse control. Attempts to reduce and wean off HIVS were made only if the dietary program showed sufficient oral intake or hyperphagia (more than 50% above the resting energy expenditure) for a period of more than 1–2 months. HIVS requirements were adjusted to individual needs, and the number of infusions per week was gradually reduced in cases of nutritional status stability, or increased to the previous level in cases of weight loss associated with a decline in the blood albumin level [76]. Although this study showed the current nutritional management in real life, it considered BMI, oral intake, and albumin levels as nutritional markers, which are not reliable for nutritional assessment in patients with CIF.

In our center, most of these patients are dependent on HIVS for a long time, or even lifelong, depending on the characteristics of the underlying disease. The first step to determine the most suitable nutritional strategy in patients with intestinal dysmotility is to perform a comprehensive nutritional assessment. This should include the same parameters as described for SBS/fistula patients, but also an exhaustive interview to identify symptoms and types of food tolerated. Oral intake is influenced by the presence of GI symptoms, so some patients receive small meals for comfort. The general recommendation is to avoid high-fat, high-residue meals that could delay gastric emptying, and simple sugar/hyperosmolar solutions (oral supplements) that could worsen distension, bloating, or abdominal pain. Enteral feeding is not used, as it has poor tolerance.

#### 2.3.4. Intestinal Transplant

##### Ideal Management

ITx has emerged as the last step in the treatment of CIF patients and should be offered to patients with irreversible intestinal failure who are not candidates for or have a poor response to AGIRS or sGLP2, and who can no longer be sustained on HIVS due to the development of major complications associated with this support.

ITx is the most complex step, because it requires integrated MIDT management in the pre-transplant and post-transplant settings; it has evolved over the past few decades due to advances in immunological knowledge and immunosuppressive drugs, as well as improvements in surgical techniques, but fundamentally due to MIDT work. The indications for ITx have been developed and established with the participation of authors, mainly from developed countries, who generally have access to all of the therapeutic strategies required to manage those patients. Those indications have been included as main listing criteria in most countries with organized donation and transplantation services, such as UNOS (US), ONT (Spain), or INCUCAI (Argentina). The advances in the management of HIVS, nutritional support, and surgical and medical management stated in the previous paragraphs clarify the reasons beyond the reduction in IFALD and the progressive increase in CRVT, becoming the leading indication for ITx.

##### Real-Life Management

According to international guidelines, consensus, and recommendations, CIF patients are candidates for ITx if they remain dependent on HIVS and develop long-term complications associated with it. However, in real life, many countries do not have the ability to offer HIVS programs and are forced to transplant earlier and without the recommended indications.

The International Intestinal Transplant Registry (IITR) was established in 1985 and has received voluntary reports of 4745 ITxs from 98 participating centers, including 2140 isolated ITxs, 1365 combined liver/ITxs, 996 MVTxs, and 244 modified MVTxs; 165 were retransplants. The IITR also presented survival data, with the overall number of reported survivors being 2133. Patient survival has shown progressive improvement over the years despite the reduction in volume. It should be noted that most cases are currently handled by centers with the greatest experience in each region. Therefore, short-term results continue to improve (with first-year graft survival over 75%), and although long-term results remain stagnant, individual large-volume centers have reported 5- and 10-year graft survival rates beyond 60%, highlighting the impact of volume and experience on outcomes [134].

A recent manuscript by L. Ceulemans et al. reports the current worldwide experience of using living donors for intestinal transplant recipients, with the greatest experience in Asian countries (accounting for approximately 20% of the total regional ITx experience) [135].

The last report of the WHO Global Observatory on Donation and Transplantation showed that 157,494 transplants were performed in 2022. Among them, 170 were intestinal transplants (0.1%). The US and UK were the countries with the greatest activity (82 and 24 ITxs, respectively). China and Iran were the most active Asian countries, with 15 and 9 ITxs, respectively, and Argentina was the only country from Latin America with 1 ITx [136].

Unfortunately, the lack of enough experts in the field, the lack of financial support to ensure access to all of the necessary therapies, and the high costs of building appropriate teams at local centers or sending patients abroad remain the most significant barriers to be overcome in the near future in order to sustain the minimal standards to provide adequate comprehensive care and results when ITx becomes an indication for CIF patients worldwide.

The impacts of volume and access will negatively impact on professional expertise. To perform at least 10 ITxs/year, including both adults and children, should be a possible benchmark to be considered and recommended by the experts in the field.

## 3. Conclusions

CIF is a complex and heterogeneous disabling disease that requires a MIDT for its management and imposes a high cost on the health system. HIVS, surgical–medical rehabilitation, and transplant are the four steps on which the successful management of CIF is based. In the past 20 years, a great number of recommendations have been published for each of these steps; however, these recommendations and guidelines are made by international societies whose members mostly work at specialized centers located in developed countries.

In real life, the access to these treatment steps is not the same in all countries of the world. The lack of dedicated MIDTs, different social environments, multifractionated health systems, and the lack of long-term plans and commitments due to frequent and non-complementary political changes (especially in low- and middle-income countries) make the implementation of such therapies unpredictable or unfeasible. These issues are not unique to underdeveloped regions; they apply to developing regions as well. The most common scenario for patient care depends solely on their chance of finding the right physician who will take care of them for life, without adequate economic recognition to allow for professional growth or team development.

In this paper, we have shown the differences that exist between the recommendations and the real-world management for each step of adult CIF treatment, even in countries on the same continent.

To help solve some of these problems, referral centers with the ability to offer the four steps of treatment should establish networks with those centers that cannot offer them. These networks could be national, continental, or even global, allowing them to homogenize the management and generate evidence while considering regional customs and habits.

Finally, the work of international societies is essential for the creation of regional registries that allow us to understand the limitations in the diagnosis, treatment, and follow-up of adult CIF patients in each region, developing their own recommendations according to their possibilities and limitations.

## Figures and Tables

**Table 1:** Drugs used for the treatment of SBS and fistula [89,90].

Effect	Drug	Dose
Antimotility	Loperamide	2–4 mg 30 min before meals and bedtime. In anatomy type 1 or 2, ≥32 mg/day
	Diphenoxylate/atropine	2.5–5 mg up to every 6 h
	Codeine	15–60 mg 4 times/day
	Opium tincture	0.3–2.0 mL 4 times/day
	Clonidine	0.3 mg/day
Antisecretory	Proton-pump inhibitors	20–40 mg 2 times/day
	Histamine 2 receptor antagonists	150–300 mg/day
	Octreotide	50–250 μg 3–4 times/day subcutaneously
	Cholestyramine	4 g up to 3 times/day
	Pancreatic enzymes	30,000–40,000 IU of lipase before major meals
Antibiotics	Metronidazole	500 mg 3 times/day
	Rifaximin	550 mg 2 times/day
	Tetracycline	250 mg/day
	Amoxicillin-clavulanate	500 mg 3 times/day
	Ciprofloxacin	500 mg 2 times/day
	Doxycycline	100 mg 2 times/day
	Norfloxacin	400 mg/day

**Table 2:** Dietary recommendations for SBS and fistula according to anatomy type.

Anatomy Type 1	Anatomy Type 2 and Type 3
-Avoid hyperosmolar solutions, control simple sugars, avoid high concentrations of salt. -Include starch-containing food. -Control lactose (less than 20 g/day).	-Control simple sugars. -Avoid oxalate consumption. -Select the type of fiber according to symptoms. -Low-fat diet.
